# The atherogenic index of plasma and carotid atherosclerosis in a community population: a population-based cohort study in China

**DOI:** 10.1186/s12933-023-01839-y

**Published:** 2023-05-27

**Authors:** Qin Huang, Zeyu Liu, Minping Wei, Qing Huang, Jie Feng, Zunjing Liu, Jian Xia

**Affiliations:** 1 Department of Neurology, Xiangya Hospital, Central South University, No.87, Xiangya Road, Changsha, 410008 Hunan China; 2 Department of Neurology, Peking University people’s hospital, Beijing, China; 3 Clinical Research Center for Cerebrovascular Disease of Hunan Province, Central South University, Changsha, China; 4 National Clinical Research Center for Geriatric Disorders, Xiangya Hospital, Central South University, Changsha, China

**Keywords:** Atherogenic index of plasma, Carotid atherosclerosis, Carotid intima-media thickness, Carotid plaque

## Abstract

**Background:**

The atherogenic index of plasma (AIP) is an important alternative metabolic biomarker of atherosclerosis and cardiovascular diseases. Nevertheless, the correlation between the AIP and carotid atherosclerosis is unknown among the general population.

**Methods:**

A total of 52,380 community residents, aged ≥ 40 years who underwentcervical vascular ultrasound from December 2017 to December 2020 in Hunan China, were selected for retrospective analysis. The AIP was calculated as a logarithmically converted ratio of triglycerides (TG) to high-density lipoprotein-cholesterol (HDL-C). The participants were divided into AIP quartile groups (Q1-Q4). Logistic regression models and restricted cubic spline analyses were used to examine the association of the AIP with carotid atherosclerosis. Stratified analyses were applied to control for confounding factors. The incremental predictive value of the AIP was further assessed.

**Results:**

After adjusting for traditional risk factors, an increased AIP was associated with a higher rate of carotid atherosclerosis (CA), increased carotid intima-media thickness (CIMT), and plaques [odds ratio, OR (95% confidence interval, CI): 1.06 (1.04, 1.08), 1.07 (1.05, 1.09), and 1.04 (1.02, 1.06) per 1-SD increase in the AIP, respectively]. Compared with those participants in the quartile 1 group, those in the quartile 4 group had a greater risk of CA [OR 1.18, 95% CI (1.12, 1.25)], increased CIMT [OR 1.20, 95% CI (1.13, 1.26)], and plaques [OR 1.13, 95% CI (1.06, 1.19)]. However, we did not observe an association between the AIP and stenosis [0.97 (0.77, 1.23), p for trend = 0.758]. Restricted cubic spline analyses also showed a cumulative increase in the risk of CA, increased CIMT, and plaques but not stenosis severity (> 50%) with an increase of the AIP. Subgroup analyses showed that a more significant association between the AIP and the prevalence of increased CA was detected in younger subjects (aged < 60 years) with a body mass index (BMI) of ≥ 24 and fewer comorbidities. Additionally, the AIP provided incremental predictive capacity over established risk factors for CA, as shown by an improvement in the net reclassification index (NRI) and integrated discrimination index (IDI) (all P < 0.05).

**Conclusions:**

An elevated AIP in a community-based population is associated with a higher rate of CA. the AIP could serve as a potential biomarker for CA risk assessment.

**Supplementary Information:**

The online version contains supplementary material available at 10.1186/s12933-023-01839-y.

## Introduction

According to the Global Burden of Diseases, Injuries, and Risk Factor Study 2019, cardiovascular diseases (CVDs), principally stroke and ischaemic heart disease (IHD), are a major contributor to disability and the leading cause of premature mortality globally [1, 2]. Atherosclerosis, the main pathological process of most CVDs [3], may start early in life and remain latent and asymptomatic for long periods before progressing into its advanced stages [4, 5]. Carotid atherosclerosis (CA), a major and potentially preventable cause of cerebrovascular disease [6], is a reflector of generalized atherosclerosis and can be assessed noninvasively with carotid ultrasonography [7]. Carotid intima-media thickness (CIMT), carotid plaques, stenosis, and occlusion, as detected by carotid ultrasound, have been shown to predict CVD risk in multiple studies [8–12]. Worldwide, approximately 28% of individuals (over one billion people) in the general population in 2020 had an abnormal CIMT of 1.0 mm and above [13]. Approximately 21% of people (816 million people) had carotid plaque and 1.5% (58 million) had carotid stenosis [13]. The large number of people living with CIMT, carotid plaque or stenosis is indicative of a future considerable burden of CVD and a major public health concern worldwide. There is an urgent need for early detection of CA among the general population, as well as for the determination of potential biomarkers and the implementation of preventive measures, considering the significant and increasing burden of atherosclerosis on individuals, families, and health-care systems.

In recent years, diverse affordable tools have been proposed as meaningful biomarkers to predict atherosclerosis and CVDs. The plasma lipid profile including triglycerides (TG), total cholesterol (TC), high-density lipoprotein cholesterol (HDL-C), and low density lipoprotein cholesterol (LDL-C) has been recognized as a key risk factor and predictor for atherosclerotic CVDs [14]. Dyslipidaemia, characterized by lipid metabolism disorders involving abnormally elevated plasma levels of lipids, is associated with the development of atherosclerosis and is assumed to be an important treatment target [15]. LDL-C is a key target for intervention for the primary and secondary prevention of atherosclerotic CVDs [16]. However, substantial residual risk persists with the majority of predicted first and recurrent CVD events not being averted despite reductions in LDL-C levels to the recommended levels [17]. Hypertriglyceridaemia, frequently associated with concomitant lipoprotein alterations such as decreased HDL-C [18], may be an important contributor to this residual risk [14]. The atherogenic index of plasma (AIP), calculated as a logarithmically converted ratio of TG to HDL-C, has been used to identify atherogenic dyslipidaemia and insulin resistance based on a positive association with cholesterol esterification rates, lipoprotein particle size, and remnant lipoproteinaemia [19, 20]. Previous studies have shown that AIP not only accurately represents the link between atherogenic and protective lipoproteins but is also implemented as a robust biomarker to prognosticate atherogenicity and CVD events [21–23]. In addition, AIP beyond the traditional risk factors has been suggested as an effective, low-cost, quick, specific, noninvasive mass screening method to identify patients who are at a high risk of CVD events [24] and to predict the rapid progression of coronary atherosclerosis [25]. However, to our knowledge, thus, limited studies have examined the association between AIP and carotid atherosclerosis, as well as CIMT, carotid plaques, and stenosis severity, especially in apparently healthy populations. Early identification of individuals with carotid atherosclerosis is of great importance for preventing their progression to advanced stages, including cardiovascular diseases.

Therefore, to fill this knowledge gap, we conducted a large cross-sectional study based on 52,380 community residents to examine the relationship between AIP and carotid atherosclerosis, and to identify the possible characteristic population in which AIP is highly associated with the carotid atherosclerosis by conducting subgroup analyses.

## Methods

### Study participants

Participants were from the China Stroke High-risk Population Screening and Intervention Program (CSHPSIP) in Hunan Province, China from 2017 to 2020. The CSHPSIP was an ongoing population-based project that enrolled community-dwelling adults aged ≥ 40 years and who lived in the project sites for more than 6 months and was organized by the Stroke Prevention and Control Steeling Committee of the National Health Commission and Centers for Disease Control and Prevention [26]. Thirteen cities with 26 communities (13 urban and 13 rural areas) were selected in proportion to the local population size and the numbers of communities were surveyed with a 2-stage stratified cluster randomized sampling design. A total of 133,489 participants received a face-to-face survey between 2017 and 2020 in Hunan, China. A total of 53,222 subjects received cervical vascular ultrasound according to the screening plan. We excluded participants with incomplete data (n = 842), and 52,380 participants with cervical vascular ultrasound examination were eligible for inclusion in the present study. Figure S1 depicts the participant selection and study design. This cross-sectional study followed the Strengthening the Reporting of Observational Studies in Epidemiology (STROBE) reporting guidelines [27]. Written informed consent was obtained from all participants before entering the study.

### Data collection

Data on sociodemographic characteristics (sex, age, XXXaterial status, and educational level), lifestyle risk factors (smoking status, alcohol consumption, and physical activities), and personal and self-reported family medical history (hypertension, diabetes mellitus, stroke, and coronary heart disease) were collected by a face-to-face interviewer-administered questionnaire. Hypertension was defined as SBP ≥ 140 mmHg and/or DBP ≥ 90 mmHg, self-reported hypertension or use of antihypertension medications [28, 29]. Diabetes mellitus was defined as self-reported diabetes mellitus, use of oral hypoglycaemic agents or insulin injections, fasting plasma glucose ≥ 7.0 mmol/L, or nonfasting glucose ≥ 11.1 mmol/L. Stroke was defined as self-reported stroke or the providing of a diagnosis certificate or an imaging certificate from a secondary or higher medical unit when considering suspected stroke. Physical activity was defined as regular physical exercise performed for > 1 year, > 2 times per week, and at least 30 min each time, or heavy physical labour. Smoking was defined as continuous or cumulative smoking for at least 6 months. Alcohol consumption was defined as the intake of alcoholic beverages at least 1 time per week. All subjects were invited to attend physical examinations including the measurement of height, body weight, and blood pressure, using a standard protocol [28]. Blood pressure was measured twice and the average was taken by trained professionals. Body mass index (BMI) was calculated as body weight (kg) divided by the square of height (m). Fasting venous blood samples were collected from an antecubital vein to measure TG, TC, HDL-L, and LDL-C. Participants were classified into high, medium, and low-risk groups according to the National Stroke Association’s Stroke Risk Scorecard [7, 26], which was composed of eight general risk factors for stroke including smoking, hypertension, atrial fibrillation, dyslipidaemia, diabetes mellitus, physical inactivity, overweight, and family history of stroke [28]. Subjects with at least three of these risk factors were classified into the high-risk stroke population, while the participants with less than 3 risk factors but having one of the following medical histories: diabetes, hypertension, and atrial fibrillation were defined as medium-risk group. Low risk of stroke was defined as having < 3 risk factors and without diabetes, hypertension, and atrial fibrillation [30]. The atherogenic index of plasma (AIP) was defined as the logarithm to the base 10 of the ratio of fasting plasma triglyceride (TG) (mg/dL) to high-density lipoprotein cholesterol (HDL-C) [log (TG/HDL-C)] [20, 31]. Subsequently, the participants were divided into four groups according to the quartile level of AIP: Quartile 1 (Q1), AIP < 0.25; Quartile 2 (Q2), AIP ≥ 0.25 and < 0.45; Quartile 3 (Q3), AIP ≥ 0.45 and < 0.66; and Quartile 4 (Q4), AIP ≥ 0.66.

### Carotid artery measurements

The subjects who enrolled in our study further underwent cervical artery ultrasonography examinations [7]. Carotid ultrasonography examinations were performed by qualified ultrasound technologists who received unified training before embarking on the study and were unaware of the baseline characteristics and laboratory results of the participants. All procedures were conducted according to the Mannheim consensus [32]. In each screening location, two sonographers evaluated carotid artery imaging data, and the final consistent carotid artery imaging data were recorded. Participants were examined using one of the following high-resolution B-mode ultrasound systems (Logiq 9, GE Healthcare and iU22, Philips Health care) with a 6- to 10-MHz linear-array transducer. The bilateral common carotid artery (CCA), internal carotid artery (ICA), external carotid artery (ECA), subclavian artery, and vertebral artery were examined and recorded. The region of interest for CIMT measurement was the far wall of the bilateral common carotid arteries proximal to the bifurcation, and an increased CIMT was defined as IMT ≥ 1.0 mm in either the right or the left carotid artery. Carotid plaques were defined as IMT ≥ 1.5 mm or focal structures encroaching into the arterial lumen of at least 0.5 mm or 50% of the surrounding intima-media thickness value [32]. Carotid stenosis was defined as 50% or more stenosis, including occlusion [5]. Participants with increased CIMT, plaques or carotid stenosis were defined as having carotid atherosclerosis [33].

### Statistical analysis

The baseline characteristics of study participants were described as mean (standard deviation) or median (interquartile range) depending on the variable distribution for continuous variables, and categorical variables were presented as count (proportion). These characteristics were compared across the AIP quartiles using analysis of variance (ANOVA) for normally distributed data, the Kruskal-Wallis test for skewed distributed data, and the chi-square test for categorical variables. The association of AIP with CA, CIMT, carotid plaques, and stenosis was evaluated with logistic regression models and expressed as the odds ratio (OR) and 95% confidence interval (CI). For each outcome of interest, three logistic models adjusted for known traditional atherosclerosis risk factors were used to study the relationship between AIP and the observed outcome. The three models included (i) Model 1, an unadjusted model; (ii) Model 2, adjusted for age, sex, education, smoking, drinking, and physical activity; (iii) Model 3, adjusted for sex, age, education, smoking, drinking, physical activity, BMI, SBP, DBP, TC, LDL-C, history of diseases including cerebrovascular disease, hypertension, and diabetes, family history of diseases including hypertension, stroke, coronary heart disease and diabetes; and (iiii) Model 4, adjusted for sex, age, education, smoking, drinking, physical activity, BMI, SBP, DBP, TC, LDL-C, history of diseases including cerebrovascular disease, hypertension, and diabetes, family history of diseases including hypertension, stroke, coronary heart disease and diabetes, lipid-lowering drugs, antihypertensive drugs, and hypoglycemic drugs. P for trends were computed using the AIP quartile as the ordinal variable. A restricted cubic spline model (adjusted for the variables in Model 4) of the odds ratios for CA, CIMT, and carotid plaques as continuous variables on a logarithmic scale was plotted to examine their association with AIP. Furthermore, stratified analyses were conducted to assess whether the potential covariables (age, sex, smoking, alcohol consumption, BMI, exercise, history of stroke, diabetes, hypertension, and stroke risk grading) modified the association between continuous and categorical AIP and CA, CIMT, carotid plaques, and stenosis severity. The incremental predictive value of AIP beyond conventional risk factors was evaluated by C-statistics, IDI, and NRI. A two-sided P value of < 0.05 was considered to indicate statistical significance, and all statistical analyses were performed using SPSS version 25.0 (IBM SPSS, Armonk, NY, USA) and R version 4.2.1 (R Development Core Team, Vienna, Austria).

## Results

### Baseline characteristics

A total of 52,380 participants (mean age 60.9 ± 10.9, 46.8% men) were included in this analysis. Overall, 26,910 (51.4%) participants had carotid atherosclerosis. Increased CIMT was present in 21,313 participants (40.7%). A total of 19,702 (37.6%) subjects developed carotid plaques. A total of 618 (1.2%) participants experienced carotid stenosis over 50%. Among them, 14,983 (28.6%), 30,173 (57.6%), and 3913 (7.5%) had a history of diabetes, hypertension, and stroke, respectively. A total of 10,419 (19.9%) consumed alcohol, 12,569 (24.0%) smoked, and 19,841 (37.9%) subjects had physical inactivity. Of note, a significant proportion of participants (54.0%) belonged to high-risk groups according to the National Association’s Stroke Risk Scorecard. The baseline characteristics of participants according to AIP quartile were presented in Table 1. The higher the quartile value of AIP, the lower the average age. Individuals in the highest AIP quartile were more likely to be male, to smoke, and to consume alcohol, to have a history of diabetes or hypertension, to have a higher proportion of family history and a lower proportion of stroke, to have a higher level of BMI, SBP, DBP, TC, TG, and to have a lower HDL-C level, but physical activity was similar across AIP quartiles. Compared with the subjects in the lower AIP groups, the participants in AIP Q4 had a higher proportion of lipid-lowering drugs, antihypertensive drugs, and hypoglycemic drugs. The number of individuals in high-risk groups increased with increasing AIP quartiles from Q1 to Q4, as well as the proportion of carotid atherosclerosis and increased CIMT. However, there was no difference in carotid stenosis (≥ 50%) according to the quartile of AIP.

**Table 1:** Baseline characteristics of the study participants according to quartiles of AIP (Atherogenic Index of Plasma)

Variables	Overall (n = 52,380)	Q1 (n = 13,405) < 0.25	Q2 (n = 12,512) 0.25 to 0.45	Q3 (n = 13,131) 0.45 to 0.66	Q4 (n = 13,332) ≥ 0.66	P-value
Age, years, mean (SD)	60.9 ± 10.9	61.7 ± 11.5	61.62 ± 11.00	60.92 ± 10.8	59.4 ± 10.5	< 0.0001
Male (%)	24,522 (46.8)	5659 (42.2)	5526 (44.2)	6131 (46.7)	7206 (54.1)	< 0.0001
Smoker (%)	12,569 (24.0)	2832 (21.1)	2700 (21.6)	3040 (23.2)	3997 (30.0)	< 0.0001
Drinker (%)	10,419 (19.9)	2362 (17.6)	2150 (17.2)	2471 (18.8)	3436 (25.8)	< 0.0001
Family history						
Diabetes	5408 (10.3)	1115 (8.3)	1225 (9.8)	1420 (10.8)	1648 (12.4)	< 0.0001
Hypertension	14,986 (28.6)	3425 (25.6)	3436 (27.5)	3802 (29.0)	4323 (32.4)	< 0.0001
Stroke	8258 (15.8)	1976 (14.7)	1996 (16.0)	2068 (15.7)	2227 (16.7)	< 0.0001
Coronary heart disease	5892 (11.2)	1318 (9.8)	1355 (10.8)	1524 (11.6)	1695 (12.7)	< 0.0001
Education (%)						< 0.0001
Less than senior high school	37,320 (71.2)	9838 (73.4)	9144 (73.1)	9302 (70.8)	9036 (67.8)	
Senior high school and above	15,060 (28.8)	3567 (26.6)	3368 (26.9)	3890 (29.2)	4296 (32.2)	
Material status (%)						< 0.0001
Married	48,260 (92.1)	12,233 (91.3)	11,495 (91.9)	12,113 (92.2)	12,419 (93.2)	
Others	4120 (7.9)	1172 (8.7)	1017 (8.1)	1018 (7.8)	913 (6.8)	
Physical activities (%)						0.117
Inactive	19,841 (37.9)	5044 (37.6)	4799 (38.4)	5042 (38.4)	4956 (37.2)	
Active	32,539 (62.1)	8361 (62.4)	7713 (61.6)	8089 (61.6)	8376 (62.8)	
Medical history (%)						
Diabetes	14,983 (28.6)	3063 (22.8)	3369 (26.9)	3953 (30.1)	4598 (34.5)	< 0.0001
Hypertension	30,173 (57.6)	6735 (50.2)	6970 (55.7)	7916 (60.3)	8552 (64.1)	< 0.0001
Stroke	3913 (7.5)	989 (7.4)	1084 (8.7)	1037 (7.9)	803 (6.0)	< 0.0001
Stroke risk grading (%)						< 0.0001
medium and low-risk	24,077 (46.0)	8352 (62.3)	6734 (53.8)	5525 (42.1)	3466 (26.0)	
high-risk	28,303 (54.0)	5053 (37.7)	5778 (46.2)	7606 (57.9)	9866 (74.0)	
Antihypertensive drugs (%)	15,935 (30.4)	3225 (24.1)	3814 (30.5)	4379 (33.3)	4517 (33.9)	< 0.0001
Lipoprotein-lowing drugs (%)	2884 (5.5)	474 (3.5)	659 (5.3)	877 (6.7)	874 (6.6)	< 0.0001
Hypoglycemic drugs (%)	7980 (15.2)	1608 (12.0)	1887 (15.1)	2118 (16.1)	2367 (17.8)	< 0.0001
BMI, kg/m2, mean (SD)	24.5 ± 3.3	23.2 ± 3.2	24.3 ± 3.2	24.9 ± 3.2	25.6 ± 3.2	< 0.0001
SBP, mmHg, mean (SD)	133.5 ± 18.4	131.0 ± 18.4	132.5 ± 18.1	134.4 ± 18.2	135.9 ± 18.5	< 0.0001
DBP, mmHg, mean (SD)	80.2 ± 10.5	78.3 ± 10.2	79.4 ± 10.1	80.8 ± 10.5	82.5 ± 10.8	< 0.0001
HDL-C, mmol/L, mean (SD)	1.35 ± 0.39	1.66 ± 0.40	1.38 ± 0.34	1.25 ± 0.31	1.09 ± 0.26	< 0.0001
LDL-C, mmol/L, mean (SD)	2.68 ± 0.90	2.56 ± 0.86	2.72 ± 0.89	2.77 ± 0.91	2.69 ± 0.91	< 0.0001
TC, mmol/L, mean (SD)	4.8 ± 1.1	4.6 ± 1.0	4.7 ± 1.1	4.8 ± 1.1	4.9 ± 1.1	< 0.0001
TG, mmol/L, median (IQR)	1.64 (1.13, 2.4)	0.93 (0.78, 1.12)	1.37 (1.18, 1.60)	1.93 (1.64, 2.24)	3.10 (2.52, 3.97)	< 0.0001
Vascular color ultrasound (%)						< 0.0001
No abnormalities	25,331 (48.4)	6854 (51.1)	5985 (47.8)	6268 (47.7)	6224 (46.7)	
Abnormalities	27,049 (51.6)	6551 (48.9)	6527 (52.2)	6863 (52.3)	7108 (53.3)	
Increased CIMT						< 0.0001
No	31,067 (59.3)	8290 (61.8)	7418 (59.3)	7765 (59.1)	7594 (57.0)	
Yes	21,313 (40.7)	5115 (38.2)	5094 (40.7)	5366 (40.9)	5738 (43.0)	
Carotid plaques						0.001
No	32,678 (62.4)	8552 (63.8)	7687 (61.4)	8176 (62.3)	8263 (62.0)	
Yes	19,702 (37.6)	4853 (36.2)	4825 (38.6)	4955 (37.7)	5069 (38.0)	
Stenosis (≥ 50%)						0.729
No	51,762 (98.8)	13,235 (98.7)	12,366 (98.8)	12,983 (98.9)	13,178 (98.8)	
Yes	618 (1.2)	170 (1.3)	146 (1.2)	148 (1.1)	154 (1.2)	
CA						< 0.0001
No	25,470 (48.6)	6891 (51.4)	6014 (48.1)	6307 (48.0)	6258 (46.9)	
Yes	26,910 (51.4)	6514 (48.6)	6498 (51.9)	6824 (52.0)	7074 (53.1)	

Data are summarized as number (percentage), mean ± standard deviation, or median (interquartile range)

CHD, coronary heart disease; BMI, body mass index; SBP, systolic blood pressure; DBP, diastolic blood pressure; TG, triglyceride; TC, total cholesterol; LDL-C, low-density lipoprotein cholesterol; HDL-C, high-density lipoprotein cholesterol; CA, carotid atherosclerosis; CIMT, carotid intima–media thicking

### Association of AIP with carotid atherosclerosis

**Fig. 1:**
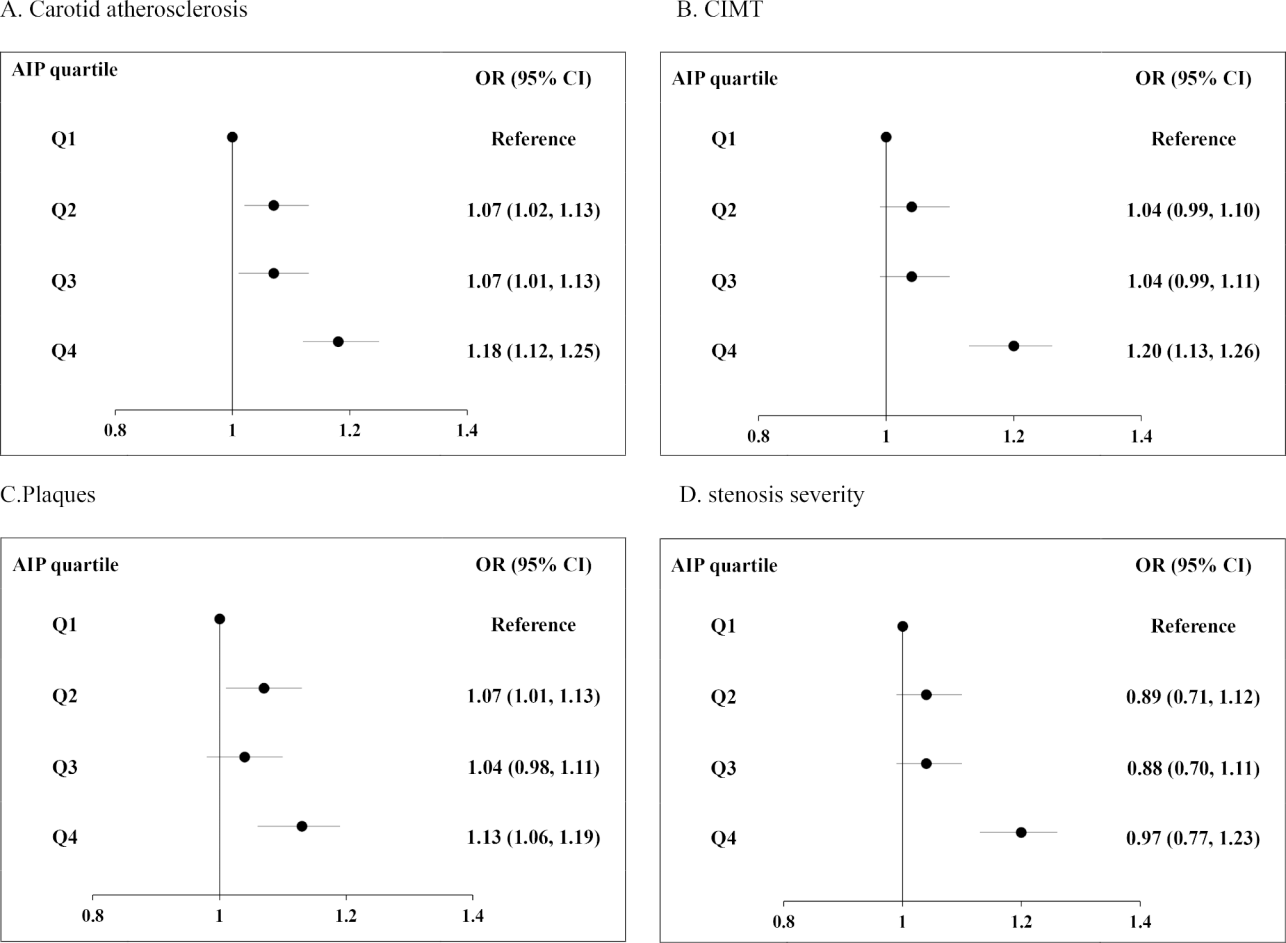
Summarized figure of ORs for (**A**) CIMT, (**B**) Carotid plaques, (**C**) Stenosis, and (**D**) CA. OR, odds ratio; CI, confidence interval. CA, carotid atherosclerosis; CIMT, carotid intima–media thickening; AIP, atherogenic Index of Plasma. Adjusted for sex, age, education, smoking, drinking, physical activity, BMI, SBP, DBP, TC, LDL-C, history of diseases including cerebrovascular disease, hypertension, and diabetes, family history of diseases including hypertension, stroke, coronary heart disease and diabetes, lipid-lowering drugs, antihypertensive drugs, and hypoglycemic drugs

**Fig. 2:**
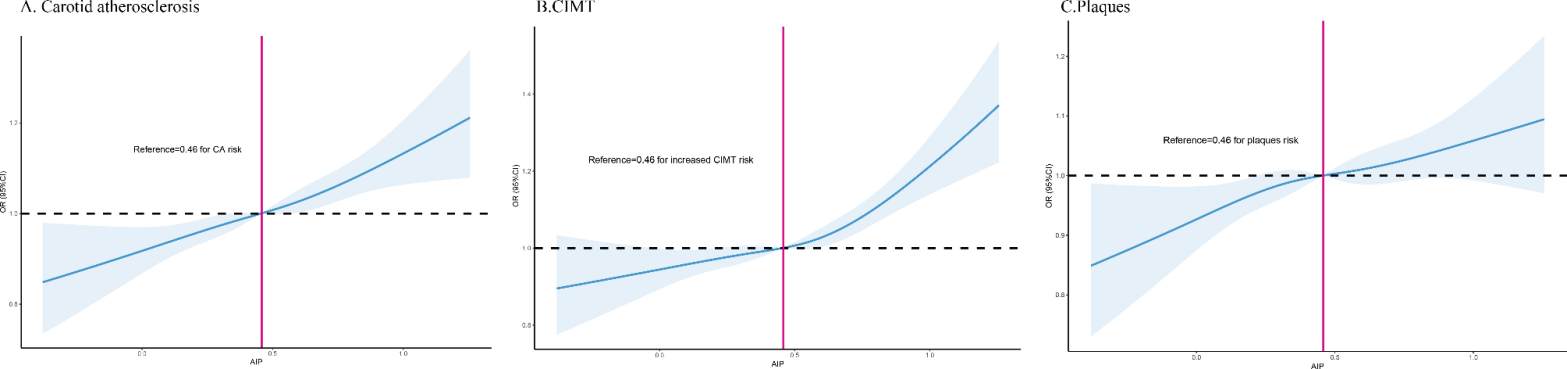
Cubic spline curves of odds ratios for CA, CMT, and plaques according to AIP values. CA, carotid atherosclerosis; CIMT, carotid intima–media thickness; AIP, atherogenic Index of Plasma. Blue lines represented references for odd ratios, and blue areas represent 95% confidence intervals. The model was adjusted for sex, age, education, smoking, drinking, physical activity, BMI, SBP, DBP, TC, LDL-C, history of diseases including cerebrovascular disease, hypertension, and diabetes, family history of diseases including hypertension, stroke, coronary heart disease and diabetes, lipid-lowering drugs, antihypertensive drugs, and hypoglycemic drugs

The results of multivariate logistic regression analysis were shown in Table 2; Fig. 1. The AIP was positively associated with the prevalence of carotid atherosclerosis, as well as increased CIMT and carotid plaques but not stenosis. In terms of the prevalence of CA, the ORs (95% CIs) were 1.07 (1.02, 1.13), 1.07 (1.01, 1.13), and 1.18 (1.12, 1.25) in AIP quartiles 2, 3, and 4, respectively, vs. quartile 1 after adjustment for traditional risk factors (p for trend < 0.0001). Compared with the lowest quartile group, the top AIP quartile was significantly associated with a higher prevalence of increased CIMT (OR: 1.20; 95% CI: 1.13–1.26, p for trend < 0.0001) and plaques (OR: 1.13; 95% CI: 1.06–1.19, p for trend < 0.0001) but not stenosis severity (OR: 0.97; 95% CI: 0.77–1.23, p for trend = 0.758). When AIP was used as a continuous variable, it was significantly associated with the prevalence of CA (OR: 1.06; 95% CI: 1.04–1.08), increased CIMT (OR: 1.07; 95% CI: 1.05–1.09), carotid plaques (OR: 1.04; 95% CI: 1.02–1.06) but not with stenosis severity (OR: 0.94; 95% CI: 0.86–1.02). the multivariable adjusted restricted cubic spline analysis, which evaluated the linear association of AIP across continuous distribution and CA, also demonstrated a significantly higher prevalence of CA, increased CIMT, and carotid plaques at higher levels of AIP (Fig. 2). The prevalence of CA, increased CIMT, and plaques increased rapidly for an AIP above 0.46 (p for linearity < 0.05).

**Table 2:** Odds ratios and 95% CIs for the association of AIP with CA, CMT, plaques and stenosis

	AIP (95% CIs)				Per SD^a^	P for trend
	Quartile 1	Quartile 2	Quartile 3	Quartile 4		
CA						
Event/total	6514/13,405	6498/12,512	6824/13,131	7074/13,332		
Incident rate (%)	48.59	51.93	51.97	53.06		
Model 1	Reference	1.14 (1.09, 1.20)	1.15 (1.09, 1.20)	1.20 (1.14, 1.26)	1.06 (1.04, 1.08)	< 0.0001
Model 2	Reference	1.17 (1.11, 1.23)	1.22 (1.16, 1.29)	1.39 (1.32, 1.46)	1.13 (1.11, 1.15)	< 0.0001
Model 3	Reference	1.09 (1.03, 1.15)	1.08 (1.02, 1.14)	1.19 (1.13, 1.26)	1.06 (1.04, 1.08)	< 0.0001
Model 4	Reference	1.07 (1.02, 1.13)	1.07 (1.01, 1.13)	1.18 (1.12, 1.25)	1.06 (1.04, 1.08)	< 0.0001
CIMT						
Event/total	5115/13,405	5094/12,512	5366/13,131	5738/13,332		
Incident rate (%)	38.16	40.71	40.87	43.04		
Model 1	Reference	1.13 (1.06, 1.17)	1.12 (1.07, 1.18)	1.23 (1.17, 1.29)	1.07 (1.05, 1.09)	< 0.0001
Model 2	Reference	1.13 (1.07, 1.19)	1.18 (1.12, 1.24)	1.38 (1.31, 1.45)	1.13 (1.11, 1.15)	< 0.0001
Model 3	Reference	1.05 (1.00, 1.10)	1.05 (1.00, 1.11)	1.21 (1.14, 1.27)	1.07 (1.04, 1.09)	< 0.0001
Model 4	Reference	1.04 (0.99, 1.10)	1.04 (0.99, 1.10)	1.20 (1.13, 1.26)	1.07 (1.05, 1.09)	< 0.0001
Carotid plaques						
Event/total	4853/13,405	4825/12,512	4955/13,131	5069/13,332		
Incident rate (%)	36.02	38.56	37.74	38.02		
Model 1	Reference	1.11 (1.05, 1.16)	1.07 (1.02, 1.12)	1.08 (1.03, 1.14)	1.02 (1.00, 1.04)	0.012
Model 2	Reference	1.14 (1.08, 1.20)	1.15 (1.09, 1.21)	1.27 (1.20, 1.34)	1.08 (1.07, 1.09)	< 0.0001
Model 3	Reference	1.08 (1.02, 1.14)	1.05 (1.00, 1.12)	1.14 (1.07, 1.20)	1.04 (1.02, 1.06)	< 0.0001
Model 4	Reference	1.07 (1.01, 1.13)	1.04 (0.98, 1.10)	1.13 (1.06, 1.19)	1.04 (1.02, 1.06)	< 0.0001
Stenosis (≥ 50%)						
Event/total	170/13,405	146/12,512	148/13,131	154/13,332		
Incident rate (%)	1.27	1.17	1.13	1.16		
Model 1	Reference	0.92 (0.74, 1.15)	0.89 (0.71, 1.11)	0.91 (0.73, 1.13)	0.92 (0.85, 1.00)	0.363
Model 2	Reference	0.96 (0.77, 1.20)	0.98 (0.78, 1.23)	1.10 (0.88, 1.38)	0.99 (0.91, 1.08)	0.409
Model 3	Reference	0.91 (0.73, 1.14)	0.90 (0.72, 1.14)	0.99 (0.79, 1.26)	0.95 (0.87, 1.03)	0.932
Model 4	Reference	0.89 (0.71, 1.12)	0.88 (0.70, 1.11)	0.97 (0.77, 1.23)	0.94 (0.86, 1.02)	0.758

Abbreviations: CA, carotid atherosclerosis; CMT, carotid intima–media thicking; AIP, Atherogenic Index of Plasma

Model 1: Unadjusted;

Model 2: Adjusted for age, sex, education, smoking, drinking, physical activity;

Model 3: Adjusted for sex, age, education, smoking, drinking, physical activity, BMI, SBP, DBP, TC, LDL-C, history of diseases including cerebrovascular disease, hypertension, and diabetes, family history of diseases including hypertension, stroke, coronary heart disease and diabetes

Model 4: Adjusted for sex, age, education, smoking, drinking, physical activity, BMI, SBP, DBP, TC, LDL-C, history of diseases including cerebrovascular disease, hypertension, and diabetes, family history of diseases including hypertension, stroke, coronary heart disease and diabetes, lipid-lowering drugs, antihypertensive drugs, and hypoglycemic drugs

^a^ Per SD, odd ratio for per SD change in AIP.

### Subgroup analysis

To explore the relationship between the AIP and CA in greater detail, we performed subgroup analyses stratified by age, sex, BMI, smoking, drinking, exercise, history of stroke, diabetes, and hypertension when the AIP was used as a continuous and categorical variable (Table 3 and Figure S2). An increased AIP (per 1-unit) was consistently related to CA, increased CIMT, and carotid plaques in various subgroups. In addition, more significant trends of AIP were observed among participants aged < 60 years with a BMI of ≥ 24. Compared to those with a history of stroke, those without a history of stroke had higher rates of CA (OR: 1.06, 95% CI: 1.04–1.08), increased CIMT (OR: 1.07, 95% CI: 1.05–1.09), and carotid plaques (OR: 1.04, 95% CI: 1.02–1.07) along with an increased AIP. Each per SD increase in the AIP was associated with approximately 1.1-fold and 1.0-fold higher rates of CA, increased CIMT, and plaques in those without and with hypertension respectively (Table 3). Further subgroup analysis of the AIP was conducted by stratifying with stroke risk screening score grading (medium and low-risk, high-risk; Table 3 and Figure S3). Consistently, the AIP was significantly associated with a higher prevalence of increased CA (OR: 1.10, 95% CI: 1.06–1.13), increased CIMT (OR: 1.09, 95% CI: 1.05–1.12), and carotid plaques (OR: 1.09, 95% CI: 1.05–1.13) in a subgroup of those in nonhigh-risk groups (Table 3). However, there was no significant association between AIP and carotid stenosis (≥ 50%) in various subgroups, which was highly consistent with the primary analysis in Table 2. Similar significant results were found when the AIP was used as a categorical variable (Figures S2 and S3).

**Table 3:** Subgroup analyses for the association of the continuous AIP with CA, CIMT, plaques and stenosis

Characteristics	Total number	OR (95% CI)			
		CA	CIMT	Plaques	Stenosis
Age					
< 60	24,954	1.14 (1.11, 1.18)	1.15 (1.11, 1.18)	1.11 (1.08, 1.15)	0.92 (0.75, 1.14)
≥ 60	27,426	0.97 (0.95, 1.00)	1.00 (0.98, 1.03)	0.98 (0.96, 1.01)	0.94 (0.85, 1.03)
Sex					
Male	24,522	1.04 (1.01, 1.08)	1.07 (1.04, 1.10)	1.01 (0.98, 1.04)	0.91 (0.82, 1.02)
Female	27,858	1.06 (1.03, 1.09)	1.06 (1.03, 1.09)	1.05 (1.02, 1.08)	0.99 (0.86, 1.13)
BMI					
< 24	23,960	1.02 (0.99, 1.05)	1.04 (1.01, 1.07)	0.99 (0.96, 1.02)	1.02 (0.91, 1.16)
≥ 24	28,420	1.10 (1.08, 1.14)	1.10 (1.07, 1.13)	1.07 (1.04, 1.10)	0.86 (0.76, 1.01)
Smoking					
Yes	14,314	1.05 (1.01, 1.09)	1.07 (1.03, 1.11)	1.02 (0.99, 1.06)	0.95 (0.83, 1.09)
No	38,066	1.06 (1.03, 1.09)	1.06 (1.04, 1.09)	1.04 (1.01, 1.06)	0.93 (0.83, 1.04)
Drinking					
Yes	10,419	1.05 (1.01, 1.10)	1.09 (1.04, 1.14)	1.03 (0.98, 1.07)	0.85 (0.71, 1.02)
No	41,961	1.06 (1.04, 1.09)	1.07 (1.04, 1.09)	1.04 (1.02, 1.06)	0.96 (0.87, 1.06)
Exercise					
Active	32,539	1.05 (1.02, 1.08)	1.06 (1.03, 1.09)	1.03 (1.00, 1.06)	0.94 (0.84, 1.06)
Inactive	19,841	1.07 (1.03, 1.10)	1.08 (1.04, 1.11)	1.04 (1.01, 1.08)	0.94 (0.82, 1.07)
History of stroke					
Yes	3913	1.02 (0.94, 1.11)	1.03 (0.95, 1.11)	0.96 (0.89, 1.03)	0.92 (0.74, 1.16)
No	48,467	1.06 (1.04, 1.08)	1.07 (1.05, 1.09)	1.04 (1.02, 1.07)	0.94 (0.86, 1.03)
History of diabetes					
Yes	14,983	1.06 (1.02, 1.10)	1.06 (1.02, 1.10)	1.04 (1.00, 1.08)	0.98 (0.86, 1.12)
No	37,397	1.06 (1.04, 1.09)	1.07 (1.05, 1.10)	1.04 (1.01, 1.06)	0.90 (0.81, 1.01)
Hypertension					
Yes	30,173	1.02 (0.99, 1.04)	1.03 (1.00, 1.05)	0.99 (0.97, 1.02)	0.94 (0.85, 1.04)
No	22,207	1.11 (1.07, 1.14)	1.12 (1.08, 1.16)	1.10 (1.06, 1.14)	0.90 (0.75, 1.07)
Stroke risk grading (%)					
medium and low-risk	24,077	1.10 (1.06, 1.13)	1.09 (1.05, 1.12)	1.09 (1.05, 1.13)	0.89 (0.74, 1.07)
high-risk	28,303	1.03 (1.00,1.06)	1.04 (1.01, 1.07)	1.01 (0.98, 1.04)	0.93 (0.84, 1.03)

Abbreviations: CA, carotid atherosclerosis; CMT, carotid intima–media thicking; AIP, Atherogenic Index of Plasma; BMI, body mass index

Adjustment for sex, age, education, smoking, drinking, physical activity, BMI, SBP, DBP, TC, LDL-C, history of diseases including cerebrovascular disease, hypertension, and diabetes, family history of diseases including hypertension, stroke, coronary heart disease and diabetes, lipid-lowering drugs, antihypertensive drugs, and hypoglycemic drugs except the corresponding stratification variable

### Incremental predictive value

To assess the predictive value of the AIP for carotid atherosclerosis, we further analysed the C-statistics, IDI, and NRI of the AIP beyond established risk factors in basic Models A, B, and C (Table 4). The C-statistics of conventional Models A, B, and C significantly improved with the addition of the AIP (all p < 0.05). Additionally, the discriminatory power and risk reclassification also appeared to be substantially better, with an NRI of 4.3% (p < 0.001) and an IDI of 0.1% (p < 0.001). Similar results were observed for increased CIMT and carotid plaques. These findings indicated that adding the AIP improved the prediction efficiency for carotid atherosclerosis.

**Table 4:** Performance of models with AIP to discriminate CA, CIMT, and plaques

Model	C statistic estimate (95% CI)	P-value	IDI	P-value	NRI	P-value
CA						
Basic model A	0.705 (0.701, 0.709)	Ref.	Ref.	Ref.	Ref.	Ref.
Basic model A + AIP	0.707 (0.703, 0.711)	< 0.001	0.003	< 0.001	0.089	< 0.001
Basic model B	0.707 (0.703, 0.710)	Ref.	Ref.	Ref.	Ref.	Ref.
Basic model B + AIP	0.708 (0.704, 0.712)	< 0.001	0.003	< 0.001	0.082	< 0.001
Basic model C	0.720 (0.716, 0.725)	Ref.	Ref.	Ref.	Ref.	Ref.
Basic model C + AIP	0.721 (0.717, 0.726)	0.036	0.001	< 0.001	0.043	< 0.001
CMT						
Basic model A	0.665 (0.661, 0.669)	Ref.	Ref.	Ref.	Ref.	Ref.
Basic model A + AIP	0.668 (0.664, 0.672)	< 0.001	0.003	< 0.001	0.088	< 0.001
Basic model B	0.667 (0.663, 0.671)	Ref.	Ref.	Ref.	Ref.	Ref.
Basic model B + AIP	0.669 (0.665, 0.673)	< 0.001	0.003	< 0.001	0.083	< 0.001
Basic model C	0.683 (0.679, 0.686)	Ref.	Ref.	Ref.	Ref.	Ref.
Basic model C + AIP	0.684 (0.680, 0.687)	0.004	0.001	< 0.001	0.046	< 0.001
Plaques						
Basic model A	0.714 (0.711, 0.718)	Ref.	Ref.	Ref.	Ref.	Ref.
Basic model A + AIP	0.715 (0.712, 0.719)	< 0.001	0.001	< 0.001	0.060	< 0.001
Basic model B	0.716 (0.713, 0.720)	Ref.	Ref.	Ref.	Ref.	Ref.
Basic model B + AIP	0.717 (0.713, 0.721)	0.003	0.001	< 0.001	0.054	< 0.001
Basic model C	0.728 (0.725, 0.733)	Ref.	Ref.	Ref.	Ref.	Ref.
Basic model C + AIP	0.729 (0.726, 0.734)	0.112	0.0002	0.035	0.032	< 0.001

Abbreviations: CA, carotid atherosclerosis; CIMT, carotid intima–media thicking; AIP, Atherogenic Index of Plasma; NRI, net reclassification index; IDI, integrated discrimination index

Basic Model A: age, sex;

Basic Model B: age, sex, education, smoking, drinking, physical activity;

Basic Model C: sex, age, education, smoking, drinking, physical activity, BMI, SBP, DBP, TC, LDL-C, history of diseases including cerebrovascular disease, hypertension, and diabetes

## Discussion

**Fig. 3:**
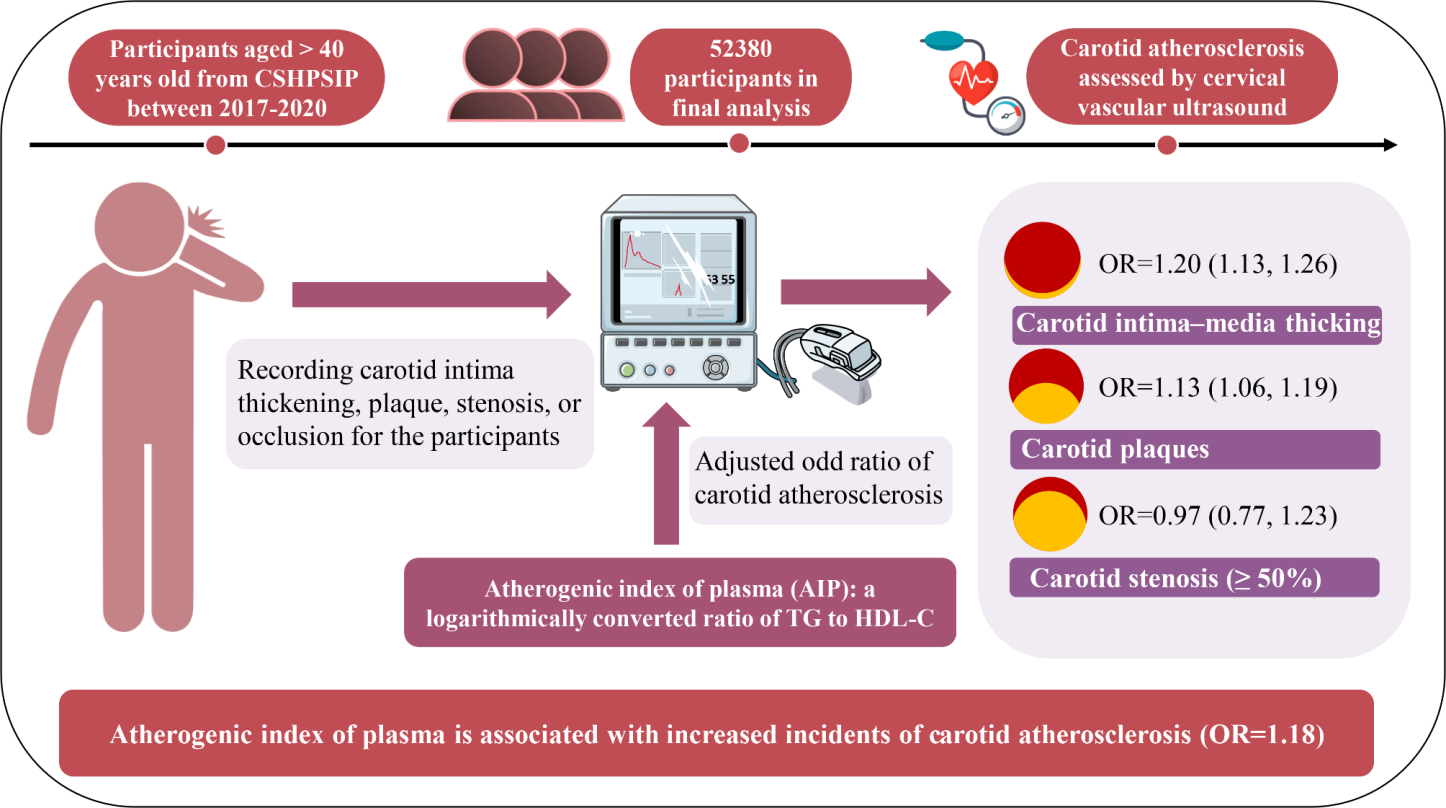
Graphical abstract of our study

Some clinical studies suggest that the AIP may be recommended as a novel predictive biomarker for cardiovascular illnesses; however, further assessment of the epidemiologic relationship between the AIP and carotid atherosclerosis is warranted, especially in a community-based population. In this analysis of a nationwide community-based cohort, we demonstrated that an elevated AIP is associated with carotid atherosclerosis, as well as increased CIMT and plaques, but not carotid stenosis (≥ 50%) among the Chinese middle-aged and elderly population. Similar findings were detected in subgroup analyses, further emphasizing the robustness of these associations. This large country-wide observational study included more than 52,000 individuals and determined that the AIP may confer an important implication for primordial prevention of atherosclerosis and become a simple yet effective tool for atherosclerotic cardiovascular risk assessment in routine practice (a structured graphical abstract is shown in Fig. 3).

Atherosclerosis develops slowly during the lifespan, and it takes decades for the clinical outcomes of atherosclerotic cardiovascular disease (ASCVD), such as myocardial, cerebral, or peripheral ischaemic syndrome, to emerge [34]. Previous studies have suggested that people with carotid atherosclerosis are at an increased risk of developing cardiovascular diseases, and the systematic detection of carotid atherosclerosis has been recommended in assessing cardiovascular risk [35, 36]. Currently, carotid atherosclerosis affects hundreds of millions of people’s health and living conditions [6]. Increased carotid intima-media thickness and carotid plaque are common disorders in the general population worldwide. Approximately one third of global cases of increased carotid intima-media thickness and carotid plaque were in the Western Pacific region in 2015 [13]. Because of the prominent role of ageing as a risk factor for carotid atherosclerosis, a larger number of people affected by carotid atherosclerosis are expected in the context of global demographic ageing. There is still a need for the identification and definition of carotid atherosclerosis in asymptomatic populations who might benefit from intervention, especially in the early stage of carotid atherosclerosis [37]. The measurement of serum biomarkers is a promising method to assist in decision-making in carotid environment burdens.

At present, a single lipid index cannot fully reflect risk of the CVDs. The AIP, correlating to lipoprotein particle size and reflecting the balance between antiatherogenic and proatherogenic particles, has been suggested as a marker for plasma atherogenicity [38] and a solid risk factor to predict CVDs by the National Cholesterol Education Program [39]. A study including 1488 adults who underwent serial coronary computed tomography angiography revealed that an increased AIP was independently associated with rapid coronary plaque progression [25]. A cross-sectional study including 340 healthy women showed a robust association between the AIP and a well-established predictive CVD biomarker (adipocyte-fatty acid binding protein) after adjustment for confounders [20]. Furthermore, a prospective study of 5538 nondiabetic coronary artery disease (CAD) patients who had received percutaneous coronary intervention demonstrated that the AIP could be used in prognostics for nondiabetic CAD patients 2 years after PCI [22]. A significant correlation with the AIP and increased CIMT was also detected during 100 Egyptian children with thalassemia major aged 6–14 years [29]. Consistently, a strong and independent correlation exists between the AIP and CIMT values in a study based on 52 male patients with ankylosing spondylitis [40]. However, most previous studies have focused on atherosclerotic vascular disease, whereas atherosclerosis in community populations has not attracted similar attention. Here, the longitudinal cohort of the Bogalusa Heart Study included 900 subjects has founded that the life-course cumulative burden of AIP was associated with arterial wall stiffening and thickening [41]. Our study based on large community residents demonstrated that the AIP was associated with the prevalence of carotid atherosclerosis, especially in the early stage of CA. These findings were in agreement with previous studies that founded a high predictive value of AIP for atherosclerosis. Of note, although a strong relationship exists between AIP and CA, CIMT, carotid plaque, the findings about the correlation between AIP and atherosclerotic stenosis is inconsistent. A study retrospectively analyzed 336 patients with acute ischemic stroke and found that AIP were closely related to the degree of carotid stenosis [42]. Another single-center study based on 31 cases of ischemic stroke showed that AIP was independently associated with symptomatic carotid stenosis [43]. However, no significant correlation was observed between AIP and asymptomatic intracranial artery stenosis in a community-based study with 5314 participants [44]. In our study, we also did not find the correlation between AIP and carotid stenosis. We speculate that the inconsistent findings about the relationship of AIP and stenosis may be related to population differences, different disease states, research methods, adjustments in multivariate models, and other factors. Our current study is an Important extension and complement of previous studies on the relationship between the AIP and atherosclerosis.

Although the AIP has been suggested as a stronger predictor of atherosclerosis and CVDs, epidemiological studies have demonstrated that the AIP has significant variability in different populations [45]. It has been established that traditional CVD risk factors including age, sex, diet, physical activity, obesity, hypertension, and diabetic mellitus, are important determinants of the AIP variability between populations [46]. In our study, we found that the positive relationship between the AIP and the rates of CA was consistent in all subgroups. A study collected 1059 patients undergoing coronary angiography and demonstrated that the AIP was independently associated with the presence and severity of acute coronary syndrome in younger patients [45]. Another study showed that the AIP was important for describing metabolic disorders and CVD risk in people < 35 years of age [47]. In the subgroup analysis, our study also showed that the effect of the AIP on the prevalence of CA was stronger in subjects aged < 60 years old than subjects aged ≥ 60 years old. In addition, previous studies suggested that there was a sex dependent difference in the association between the AIP and CVD [48, 49]. The AIP was shown to be an independent risk factor for CVDs in women [49]. Middle-aged female subjects were more likely to have central adiposity due to a reduced basal metabolic rate, and the oestrogen deprivation secondary to menopause may lead to adverse CVDs [50]. In the present study, we further found that elevated AIP was associated with a higher prevalence of CA, especially among females, which is consistent with the results in the general population [51]. In the stratified analysis, the positive associations between the AIP and CA were stronger in those without hypertension and stroke. The findings in the subgroup analysis according to the National Association’s Stroke Risk Scorecard have shown good agreement in terms of the above conclusions. One possible explanation is that the more traditional risk factors weakens the interpretation of effects and could influence the robustness of associations [24]. A previous study based on 8390 subjects suggests that AIP values of -0.3 to 0.1 are associated with low CVD risk, 0.1 to 0.24 with medium CVD risk and above 0.24 with high CVD risk [52]. Approximately 75% of enrolled participants in our study were among those in the “high risk” group according to the above values. It is perhaps relevant that the enrolled populations in our study have more concomitant diseases according to the screening plan. Thus, health programs to prevent CVDs in assessed populations are imperative.

Several potential mechanisms were proposed to elucidate the correlations between the AIP and carotid atherosclerosis. First, the AIP is positively associated with the fractional esterification rate of HDL-C, especially with small-dense LDL (sdLDL) [38]. A high proportion of sdLDL in AIP reflects the complex interactions of lipoprotein metabolism and is useful in predicting plasma atherogenicity [53]. It is small-sized, with reduced clearance from the bloodstream, more sensitive to oxidative stress, easily converted into oxidized LDL in the body, and eventually causes inflammatory responses in the sub-endothelium of blood vessels, enhanced binding to endothelial proteoglycans, and generates foam cells [38]. These properties are the initial stages of atherogenesis. Second, the AIP plays an important role in regulating the reverse cholesterol transport process, which associated with the recycling or disposal of excess cholesterol [54]. Increased AIP may mean adipocytes to store excess TG as fat, increase the accumulation of the cholesterol crystals in the inner layers of the atherosclerotic arteries, cause the narrowing and blockage of the lumen, and eventually lead to the formation of atherosclerosis.

Several limitations impact the interpretation of our findings. Some interventions such as differences in diet, race, lifestyle, and different postmenopausal and metabolic statuses might also affect AIP values. Furthermore, the study design necessitates a retrospective analysis of observational data and therefore the causative link between the AIP and CA cannot be determined. Third, the study included only Chinese participants aged 40 years and above, so the present findings should be generalized to other populations with caution. Finally, the potential mechanism of the association between the AIP and CA requires further prospective large-scale study. Despite these limitations, the well-established cohort and its relatively large size strengthen the validity of our results. Given that the AIP can be readily calculated from a the routine lipid profile, it is already available for use in clinical practice, especially using large screening programs.

## Conclusions

Elevated AIP values in community-based populations are associated with the prevalence of carotid atherosclerosis, including increased CIMT, and plaques, but not carotid stenosis severity. The association is higher in middle-aged individuals than in elderly individuals. In addtion, the relationship is stronger among individuals with overweight and with normal blood pressure. The AIP could be an effective marker for atherosclerosis and future CVD events.

### Electronic supplementary material

Below is the link to the electronic supplementary material.

**Figure S1.** Flow chart for selecting participants for analysis. AIP, atherogenic index of plasma

**Figure S2.** Subgroup analyses for the association of the quartiles of AIP with CA, CIMT, and plaques. CA, carotid atherosclerosis; CIMT, carotid intima–media thickness; AIP, atherogenic Index of Plasma. Adjustment for sex, age, education, smoking, drinking, physical activity, BMI, SBP, DBP, TC, LDL-C, history of diseases including cerebrovascular disease, hypertension, and diabetes, family history of diseases including hypertension, stroke, coronary heart disease and diabetes, lipid-lowering drugs, antihypertensive drugs, and hypoglycemic drugs except the corresponding stratification variable

**Figure S3.** The relationship between the AIP and CA, CIMT, plaques and stenosis in high-risk population and in the medium and low-risk populations. CA, carotid atherosclerosis; CIMT, carotid intima–media thickness; AIP, atherogenic Index of Plasma. The study population was stratified according to the National Stroke Association’s Stroke Risk Scorecard

## Data Availability

The datasets used and/or analyzed during the present study are available from the corresponding author on reasonable request.

## Abbreviations

AIP: Atherogenic Index of Plasma
BMI: Body mass index
SBP: Systolic blood pressure
DBP: Diastolic blood pressure
HDL-C: High-density lipoprotein-cholesterol
LDL-C: Low-density lipoprotein-cholesterol
TG: Triglycerides
TC: Total cholesterol
CA: Carotid atherosclerosis
CIMT: Carotid intima–media thickness
CVD: Cardiovascular disease
HR: Odd ratio
CI: Confidence interval
