# Correction: The atherogenic index of plasma and carotid atherosclerosis in a community population: a population-based cohort study in China

**DOI:** 10.1186/s12933-023-01977-3

**Published:** 2023-09-07

**Authors:** Qin Huang, Zeyu Liu, Minping Wei, Qing Huang, Jie Feng, Zunjing Liu, Jian Xia

**Affiliations:** 1 Department of Neurology, Xiangya Hospital, Central South University, No.87, Xiangya Road, Changsha, 410008 Hunan China; 2 Department of Neurology, Peking University people’s hospital, Beijing, China; 3 Clinical Research Center for Cerebrovascular Disease of Hunan Province, Central South University, Changsha, China; 4 National Clinical Research Center for Geriatric Disorders, Xiangya Hospital, Central South University, Changsha, China

**Correction to: Cardiovascular Diabetology (2023) 22:125** 10.1186/s12933-023-01839-y

Following publication of the original article [1], the author noticed the error in figure.

In the image of Figure 1D, the forest plot of Figure 1D does not match the corresponding odds ratio (OR) and 95% confidence interval (CI). The error occurred due to our failure to carefully inspect and match the forest plot of Figure 1D during the layout process of the article. The corresponding caption of Figure 1 should be "Summarized figure of ORs for (A) CA, (B) CIMT, (C) Plaques, and (D) Stenosis severity," rather than "Summarized figure of ORs for (A) CIMT, (B) Carotid plaques, (C) Stenosis, and (D) CA." This correction aligns with the accurate presentation of our research findings. The corrected version of Figure 1 should appear as shown below:

**Fig. 1:**
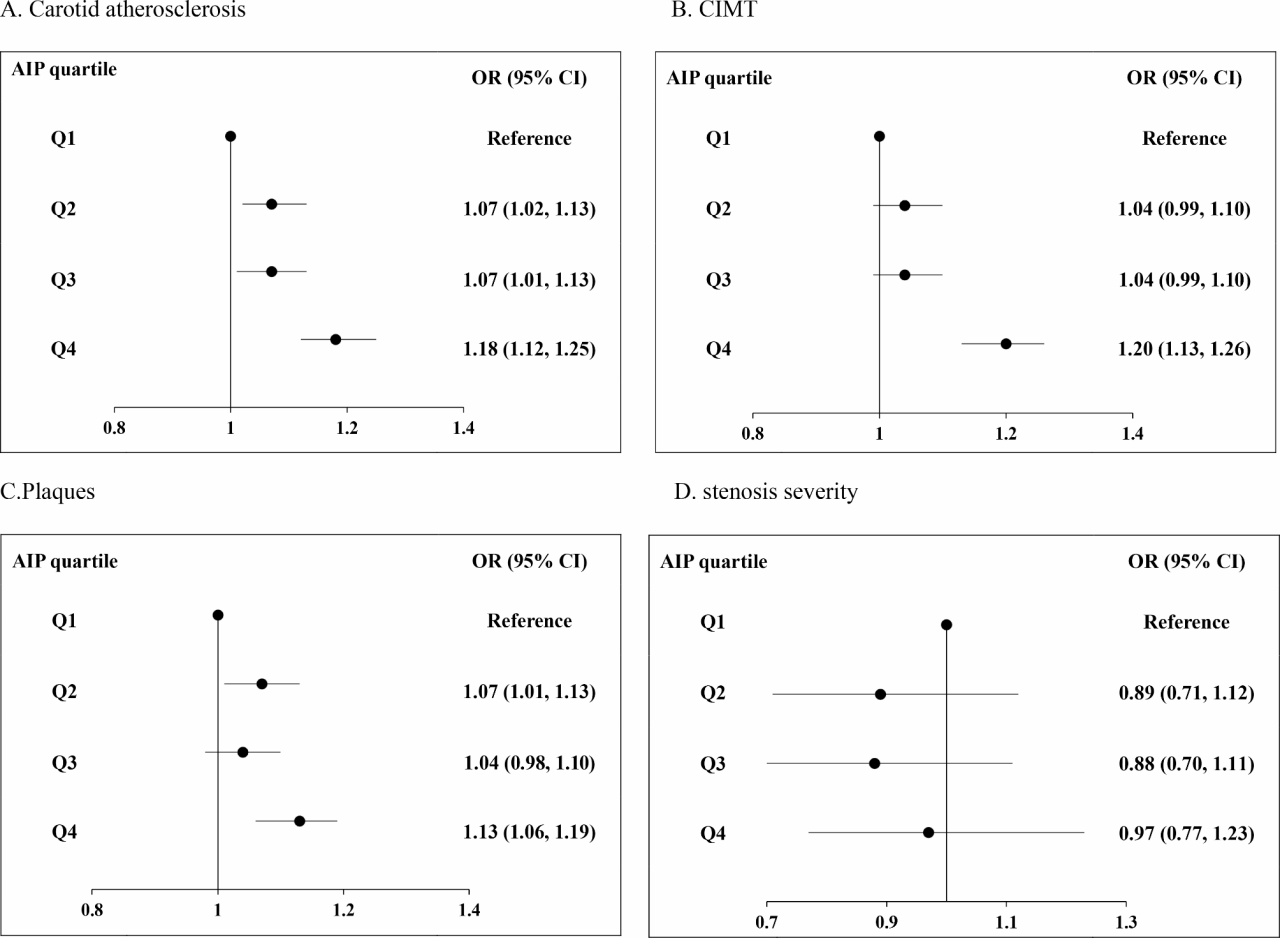
Summarized figure of ORs for (A) CA, (B) CIMT, (C) Plaques, and (D) Stenosis severity. OR, odds ratio; CI, confidence interval; CA, carotid atherosclerosis; CIMT, carotid intima?media thickening; AIP, atherogenic Index of Plasma. Adjusted for sex, age, education, smoking, drinking, physical activity, BMI, SBP, DBP, TC, LDL-C, history of diseases including cerebrovascular disease, hypertension, and diabetes, family history of diseases including hypertension, stroke, coronary heart disease and diabetes, lipid-lowering drugs, antihypertensive drugs, and hypoglycemic drugs.
