# Supplementary figures and images for: The atherogenic index of plasma and carotid atherosclerosis in a community population: a population-based cohort study in China
Source: Cardiovasc Diabetol. 2023 May 27;22:125. doi: 10.1186/s12933-023-01839-y (PMC10225098; doi:10.1186/s12933-023-01839-y)

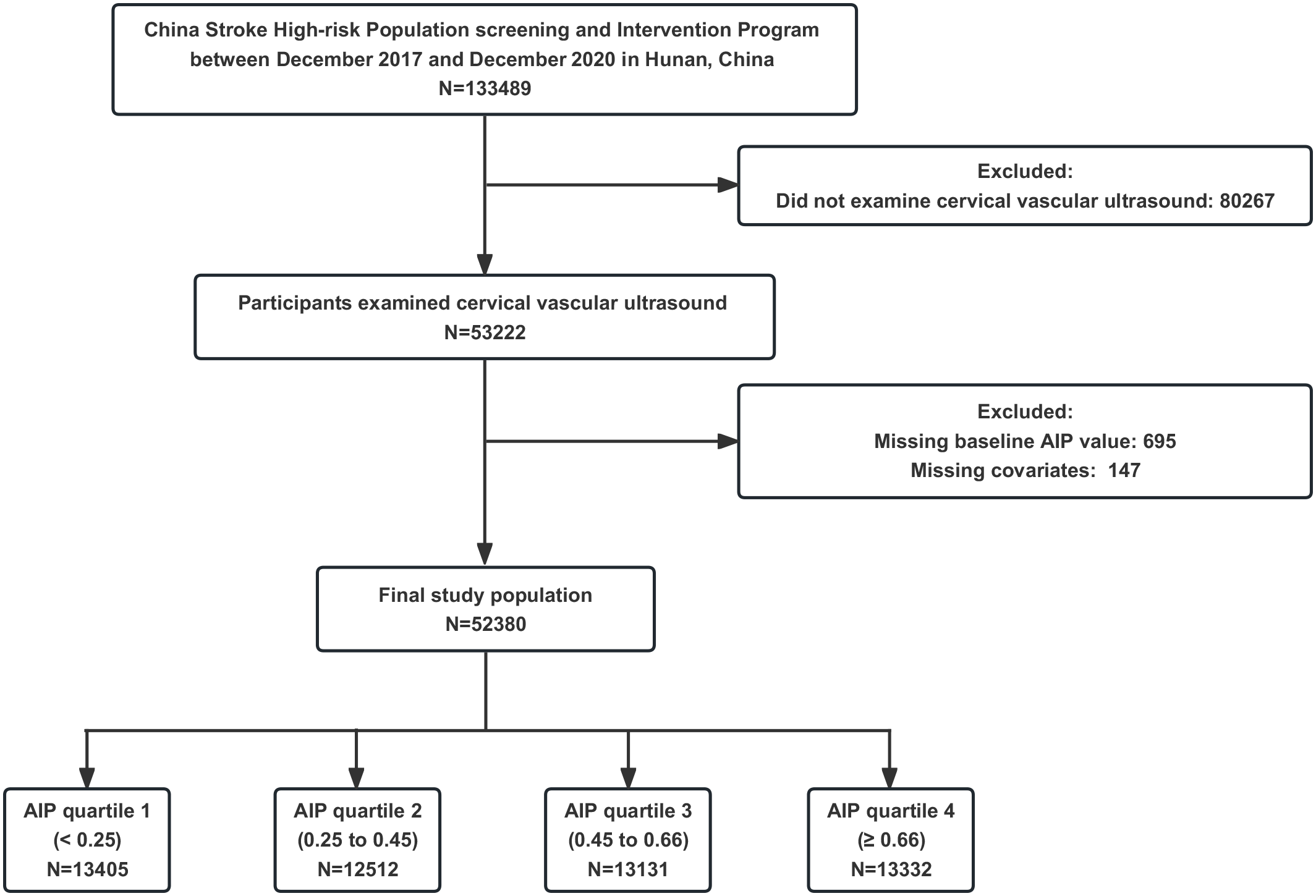

Supplement: Supplementary file 1 — Figure S1. Flow chart for selecting participants for analysis. AIP, atherogenic index of plasma [file 12933_2023_1839_MOESM1_ESM.png]

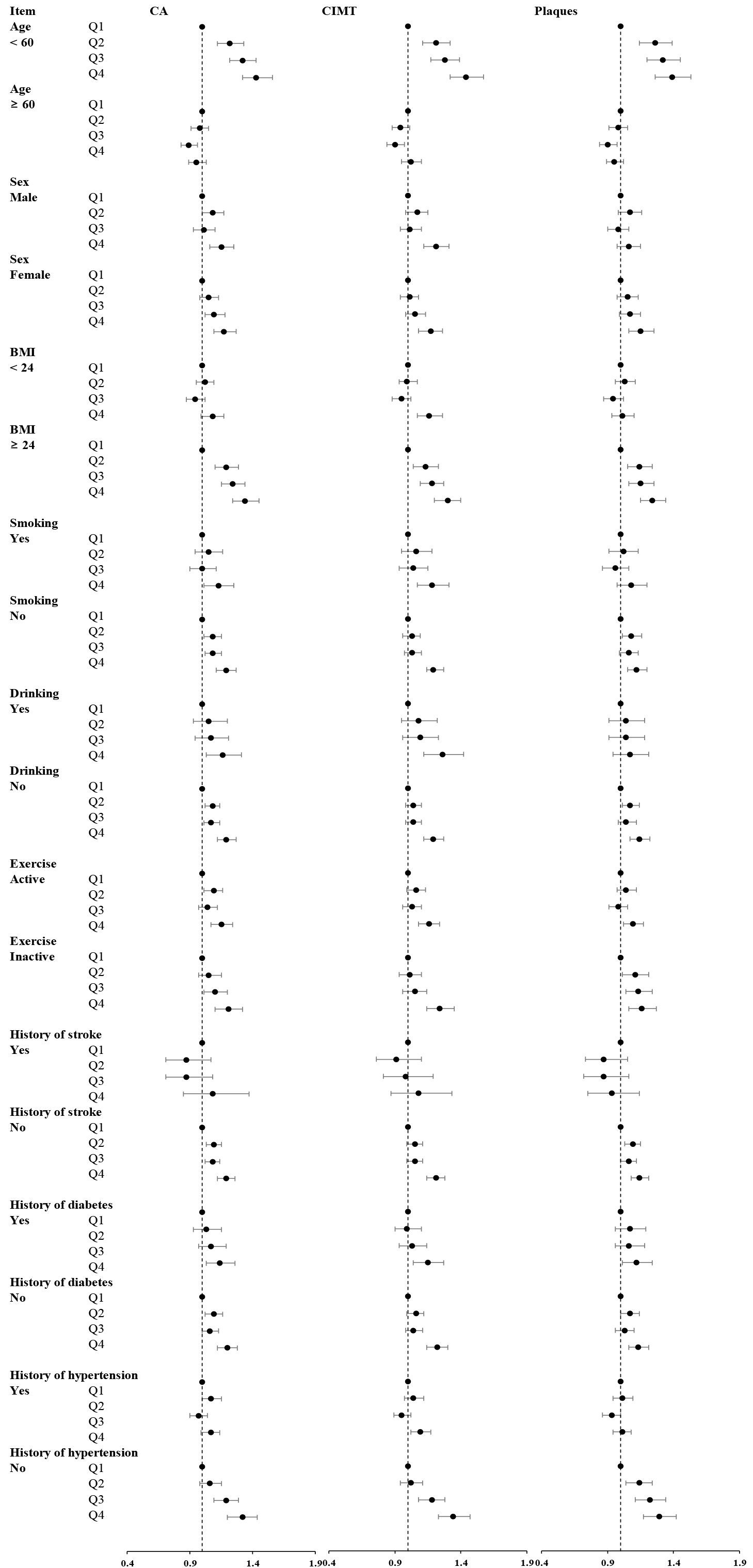

Supplement: Supplementary file 2 — Figure S2. Subgroup analyses for the association of the quartiles of AIP with CA, CIMT, and plaques. CA, carotid atherosclerosis; CIMT, carotid intima–media thickness; AIP, atherogenic Index of Plasma. Adjustment for sex, age, education, smoking, drinking, physical activity, BMI, SBP, DBP, TC, LDL-C, history of diseases including cerebrovascular disease, hypertension, and diabetes, family history of diseases including hypertension, stroke, coronary heart disease and diabetes, lipid-lowering drugs, antihypertensive drugs, and hypoglycemic drugs except the corresponding stratification variable [file 12933_2023_1839_MOESM2_ESM.png]

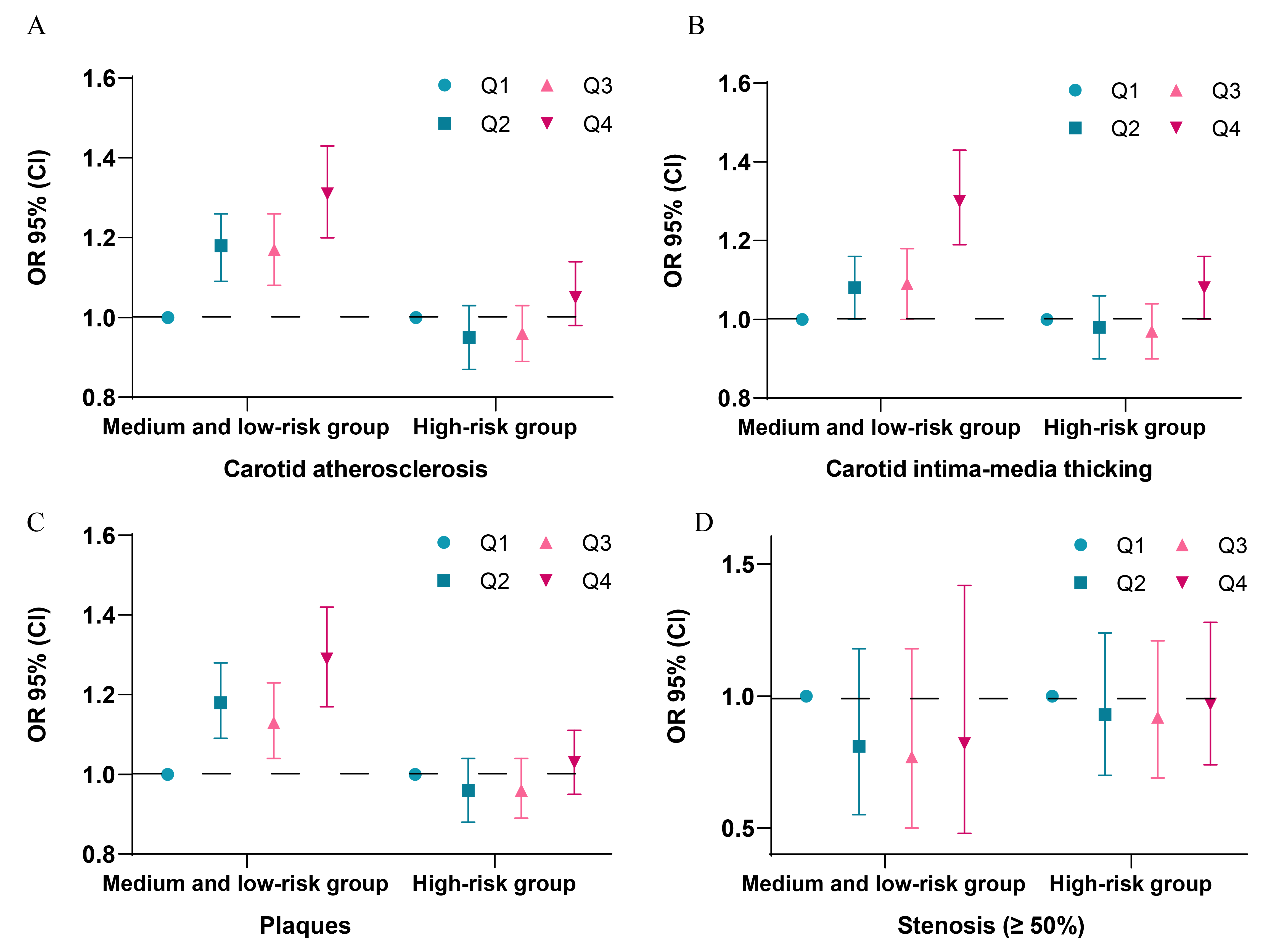

Supplement: Supplementary file 3 — Figure S3. The relationship between the AIP and CA, CIMT, plaques and stenosis in high-risk population and in the medium and low-risk populations. CA, carotid atherosclerosis; CIMT, carotid intima–media thickness; AIP, atherogenic Index of Plasma. The study population was stratified according to the National Stroke Association’s Stroke Risk Scorecard [file 12933_2023_1839_MOESM3_ESM.png]
